# Complete Genome Sequence of Roe Deer Picobirnavirus Strain PBV/roe_deer/SLO/D38-14/2014

**DOI:** 10.1128/genomeA.01329-17

**Published:** 2017-12-14

**Authors:** Urska Kuhar, Gorazd Vengust, Urska Jamnikar-Ciglenecki

**Affiliations:** a Institute of Microbiology and Parasitology, Veterinary Faculty, University of Ljubljana, Ljubljana, Slovenia; b Institute of Pathology, Wild Animals, Fish and Bees, Veterinary Faculty, University of Ljubljana, Ljubljana, Slovenia; c Institute of Food Safety, Feed and Environment, Veterinary Faculty, University of Ljubljana, Ljubljana, Slovenia

## Abstract

Picobirnaviruses (PBVs) have been detected in feces from various animal species and humans. Here, we report the complete genome sequence of the PBV/roe_deer/SLO/D38-14/2014 strain, which is the first PBV detected in roe deer, providing additional knowledge about the high diversity and host range of PBVs.

## GENOME ANNOUNCEMENT

Picobirnaviruses (PBVs) belong to the genus *Picobirnavirus*, which is the only member of the family *Picobirnaviridae*. The PBV genome is bisegmented, double-stranded RNA. The larger genome segment 1 (2.2 to 2.7 kb) has two or three open reading frames (ORFs), with the largest ORF coding for capsid protein. The smaller genome segment 2 (1.2 to 1.9 kb) has one ORF coding for the viral RNA-dependent RNA polymerase (RdRp) (1, 2). PBVs have been detected in fecal samples from humans and also from various animal species, including wildlife, suggesting a wide host range of these viruses (1–6). To our knowledge, this is the first report of PBV detected in roe deer.

A survey throughout Slovenia was performed to screen game animals, including roe deer, for the presence of enteric viruses (7). In this survey, next generation sequencing (NGS) for a complete genome determination of rotavirus A was used for sample SLO/D38-14 (8), in which a PBV was also detected.

The protocol for NGS has already been described (8). Sequenced reads were assembled into contigs by *de novo* assembly, using the SPAdes v3.1.0 (9). The contigs were compared to the GenBank nonredundant nucleotide and protein database (BLASTn, BLASTx). The BLAST searches revealed that 2 contigs belong to PBVs, representing the complete bisegmented genome sequence of the PBV/roe_deer/SLO/D38-14/2014 strain. The final genome analysis was performed using the Geneious software suite v9.1.8 (Biomatters Ltd., Auckland, New Zealand). Open reading frames were predicted with the Geneious ORF finder. Putative ORFs were subjected to BLASTp. Genome segments 1 and 2 of the PBV/roe_deer/SLO/D38-14/2014 strain were aligned with PBV genome segments deposited in GenBank. Nucleotide (nt) and amino acid (aa) sequence alignments were constructed using the MAFFT algorithm (4). Based on the alignments, the aa identities were determined.

Segment 1 of the PBV/roe_deer/SLO/D38-14/2014 strain is 2,576 nt long, with three ORFs and five conserved bases, GUAAA, at the 5′ end. The ORF1 and ORF2 code for hypothetical proteins, while the largest ORF3 codes for a capsid protein. The capsid protein aa sequence of the roe deer PBV strain is highly divergent from other PBV capsid protein sequences deposited in GenBank, as it shares only 22.3% aa identity with the most closely related PBV strain. Segment 2 of the PBV/roe_deer/SLO/D38-14/2014 strain is 1,721 nt long with five conserved bases at the 5′ and 3′ end, GUAAA and ACUGC, respectively. Segment 2 has only one ORF coding for RdRp. When comparing the RdRp coding region of the roe deer PBV and PBVs deposited in GenBank, the roe deer PBV shared 66.9% aa identity with the closest relative.

This is the first report of a PBV detected from roe deer with complete genome determination, which confirms the high diversity of PBVs and also widens the host range of these viruses.

### Accession number(s).

The complete bisegmented genome sequence of PBV strain PBV/roe_deer/SLO/D38-14/2014 has been deposited in GenBank under accession numbers MG190028 (segment 1) and MG190029 (segment 2).
